# Affinity Inequality among Serum Antibodies That Originate in Lymphoid Germinal Centers

**DOI:** 10.1371/journal.pone.0139222

**Published:** 2015-10-07

**Authors:** Myungsun Kang, Timothy J. Eisen, Ellen A. Eisen, Arup K. Chakraborty, Herman N. Eisen

**Affiliations:** 1 Department of Chemical Engineering, Massachusetts Institute of Technology, Cambridge, Massachusetts, United States of America; 2 Institute for Medical Engineering & Science, Massachusetts Institute of Technology, Cambridge, Massachusetts, United States of America; 3 Department of Biology, Massachusetts Institute of Technology, Cambridge, Massachusetts, United States of America; 4 Whitehead Institute for Biomedical Research, Massachusetts Institute of Technology, Cambridge, Massachusetts, United States of America; 5 Environmental Health Sciences, School of Public Health, University of California, Berkeley, California, United States of America; 6 Department of Physics, Massachusetts Institute of Technology, Cambridge, Massachusetts, United States of America; 7 Department of Chemistry, Massachusetts Institute of Technology, Cambridge, Massachusetts, United States of America; 8 Department of Biological Engineering, Massachusetts Institute of Technology, Cambridge, Massachusetts, United States of America; 9 Ragon Institute of the Massachusetts General Hospital, Massachusetts Institute of Technology, and Harvard Medical School, Cambridge, Massachusetts, United States of America; 10 Koch Institute for Integrative Cancer Research, Massachusetts Institute of Technology, Cambridge, Massachusetts, United States of America; Chang Gung University, TAIWAN

## Abstract

Upon natural infection with pathogens or vaccination, antibodies are produced by a process called affinity maturation. As affinity maturation ensues, average affinity values between an antibody and ligand increase with time. Purified antibodies isolated from serum are invariably heterogeneous with respect to their affinity for the ligands they bind, whether macromolecular antigens or haptens (low molecular weight approximations of epitopes on antigens). However, less is known about how the extent of this heterogeneity evolves with time during affinity maturation. To shed light on this issue, we have taken advantage of previously published data from Eisen and Siskind (1964). Using the ratio of the strongest to the weakest binding subsets as a metric of heterogeneity (or affinity inequality), we analyzed antibodies isolated from individual serum samples. The ratios were initially as high as 50-fold, and decreased over a few weeks after a single injection of small antigen doses to around unity. This decrease in the effective heterogeneity of antibody affinities with time is consistent with Darwinian evolution in the strong selection limit. By contrast, neither the average affinity nor the heterogeneity evolves much with time for high doses of antigen, as competition between clones of the same affinity is minimal.

## Introduction

The strength of bonds formed by antibodies (Abs) with antigens (Ags) is one of the critical determinants of immune responses against pathogens. Ags are generally proteins and structurally so complex that much of what was first learned about Ab-Ag bonding, and how that evolves as affinity maturation ensues, is based upon Ab binding of small molecules that closely approximate the sites on protein Ags (epitopes) that are recognized by Abs. Called haptens, these small molecules bind specifically to Ag binding sites but themselves are not immunogenic. The strengths of the bonds that haptens form with Abs extend over about a million-fold range (10^3^ or 10^4^–10^10^ M^-1^) [1,2]. This range encompasses the bond strengths measured for authentic protein Ags binding with Abs—whether monoclonal Abs or average values for heterogeneous (polyclonal) populations of purified Abs isolated from serum.

Long before Ab affinity could be measured it was known that immune sera to bacteria, red blood cells and proteins cross-reacted with structures that resembled the inciting antigen (the immunogen) and that after adsorption or precipitation of all Abs to cross-reacting (heterologous) structures, the remaining Abs could still react with the immunogen. These findings were attributed to a diversity of serum Abs that could react with different components of the complex immunogens. In 1936, however, Landsteiner and van der Scheer showed that antisera raised against an immunogen having a chemically defined epitope (azophenylsuberanilic acid) could be exhaustively adsorbed with various cross-reacting alternate forms of the epitope and the remaining Abs could still react with the homologous epitope; hence their conclusion that Abs to a singular epitope “are not entirely uniform but vary in specificity to some degree” [3].

Studies of Ab binding to haptens confirmed and extended previous work on more complex immunogens. Haptens used either to inhibit specific precipitation of Abs from antisera by haptenated Ags (hapten-inhibition) [4], or to bind directly, in absence of any Ag, to purified Abs [5], demonstrated variability in Ab binding. When ligands were added incrementally to Abs at a constant concentration in hapten inhibition experiments, the resulting binding curves were nearly always non-linear, as though apparent equilibrium constants, determined for each point, decreased with increasing concentration of the ligand. Such non-linearity was attributed to the variability inherent in the combined free energy of Abs with hapten in heterogeneous anti-arsanilate antiserum, which could be accounted for by a Gaussian error function [4]. There were misgivings about the quantitative nature of this approach because in hapten-inhibition of specific precipitation the composition of soluble complexes—of Ab, Ag, and hapten—were unknown [6]. However, Karush found that there was good agreement between hapten-Ab interactions measured directly by equilibrium dialysis and the theoretical binding curves based upon an assumed Gaussian distribution of free energy of hapten-Ab binding [7,8]. Thus binding of a hapten to a population of cognate Ab molecules isolated from serum could be characterized by two constants: i) the average bond-strength (K_0_, the mean equilibrium association constant or intrinsic affinity), and ii) an index of heterogeneity with respect to affinity (sigma, σ).

In response to most immunogens, Abs made initially have low affinity and those made later have progressively higher affinity [9,10]. This progression, or affinity maturation, arises from events that take place in germinal centers (GC), small clusters of cells in secondary lymphoid tissues including lymph nodes, spleen, and Peyer’s patches on intestinal mucosae [11]. Much of what we know about how processes in GCs lead to higher affinity Abs was learned subsequent to the first descriptions of affinity maturation. Each GC is formed by a few antigen (Ag)-stimulated naïve B cells [12] which, in GC, express the antigen-activated cytidine deaminase, AID, that causes mutations in the variable regions of the H and L chains of the Ab expressed by that B cell [13]. The diverse population of B cells thus generated express different Ag-binding receptors (BCR). These cells are then selected against the antigen, which is displayed on follicular dendritic cells (FDC) in the GC [14–16]. Cells with BCRs that have high affinity for Ag bind it more readily than those with low affinity BCRs and are thus more likely to receive survival signals [17]. BCRs are also endocytic receptors [18]. Hence, cells with high affinity BCRs are more capable than those with low affinity BCRs to endocytose the Ag and present it as peptide-MHC-II complexes. These peptide-MHC-II complexes can engage with T cell receptors on the surface of cognate CD4 helper T cells in the GC [19]. The different B cells compete with each other for limiting numbers of T helper cells; B cells that internalize more antigens have a competitive advantage. Receipt of signals from T helper cells has been shown to be the key gatekeeper of B cell survival [20]. Inability to bind to Ag sufficiently strongly or receive T cell help results in apoptosis [21]. A few selected B cells emerge from the GC and differentiate into memory cells and antibody-secreting plasma cells, but most are recycled for further rounds of mutation and selection [22]. Thus, in vaccinated or infected individuals, Darwinian evolution occurs in a relatively short time-scale to generate antibodies with increasingly higher affinity as time ensues.

Advances in microscopy of live cells in intact lymph nodes have provided remarkable visualization of the cellular dynamics underlying the evolutionary events in GC [17,20,23]. Implicit in these observations, and in some of the mathematical models that characterize GC reactions, is an expectation that the Abs produced in a given immune response evolve over time to become relatively homogeneous and of high affinity [24–27]. Previously, however, affinity heterogeneity was found to increase over time, not to decrease [28]. Resolving this disparity is of interest as the extent of heterogeneity may have implications for the evolution of cross-reactive antibodies against highly mutable and persistent virus infections such as HIV-1 and HCV.

To examine the disparity between expected and observed changes in the heterogeneity of affinity of Abs undergoing affinity maturation, we re-analyzed previously published data of Eisen and Siskind [28] on the binding of haptens by serum Abs. Our analysis is based on affinity measurements of purified Abs obtained from rabbit sera. The protocol from the original paper collected sera over several weeks after dosing with a range of the hapten-bearing immunogens and characterized each collected population by average equilibrium constant (K_0_) and sips heterogeneity index (a). In view of the overall approximately million-fold range in intrinsic affinity values measured for Ab-hapten and Ab-Ag reactions in general, our re-analysis addresses three specific questions. 1. How large is the diversity of Ab affinities, henceforth referred to as affinity inequality, in bleeds from individual animals or serum pools from a few individuals (rabbits)? 2. Does affinity inequality change over time after initiation of the response to the injected Ag? 3. To what extent is inequality affected by the quantity of Ag introduced?

To measure affinity inequality, we have relied primarily on affinity ratios between the strongest binding 5% and the weakest binding 5% of each purified Ab population. In response to relatively small doses of the immunogen, the heterogeneity indices have been found to increase over time [29,30]. Eisen and Siskind (26) originally interpreted their observation that heterogeneity increases over time to suggest that maintenance of Ab heterogeneity during the immune response is evolutionarily advantageous. But, we now show here, at low Ag dose, the extent of heterogeneity changes more slowly than the increase in the average affinity. As a result, the effective affinity inequality decreases. At high doses of Ag, however, affinity inequality is greater and more persistent. This finding suggests that, at low Ag dose, the Darwinian evolution process of affinity maturation is in the strong selection limit, while this is not the case when Ag dose is high.

## Methods

The data we analyze are drawn from studies in which rabbits were injected with “haptenated” proteins 2, 4- dinitrophenyl (DNP)-bovine-γ-globulin. The quantities of Ag administered varied from 5mg to 250mg given as a single injection via footpads. Hapten-specific Abs were precipitated (or adsorbed) from serum by hapten-protein conjugates in which the protein component differed from (and did not cross-react with) the protein in the immunogen. Purified Abs recovered from the precipitates (or adsorbates) corresponded generally to ≈ 30–90% of total precipitable Abs in sera. Binding of haptens, ε-DNP-L-lysine, at various concentrations by fixed amounts of purified Abs were measured by equilibrium dialysis or fluorescence energy transfer (at 2–30°C).

The average equilibrium constants (K_0_) were obtained as the reciprocal of the free ligand concentration when half the Ab binding sites were occupied [4,31]. From titrations of the hapten-Ab binding, an index of heterogeneity with respect to affinity could be obtained from the generalized adsorption isotherm [6,32]

$$\frac{r}{n}\;=\;\frac{{{(K}_{0}c)}^{a}}{1+{\left(K_{0}c\right)}^{a}}$$ (1)

or more conveniently when expressed in alternative form [33] as

$$\text{log}\;\left(\frac{r}{n-r}\right)\;=\;a\;\text{log}\left(K_{0}\right)+a\;\text{log}\left(c\right)$$ (2)

where r is mols hapten bound per Ab molecule, n is the number of binding sites per Ab molecule of the IgG type (2), c is the free ligand concentration, and *a* is an index of heterogeneity with respect to K_0_ (affinity). By plotting $\text{log}\;\left(\frac{r}{n-r}\right)$ vs. log(*c*), the data fall on a straight line whose slope is *a*, the Sips index of heterogeneity with respect to K_0_.

The Sips and Gaussian probability distributions are virtually congruent [32] and affinity inequality was measured by converting values for the Sips heterogeneity index (*a*) to sigma (σ), the corresponding index of heterogeneity of the Gaussian distribution. The conversion was based upon the assumption that variations in ΔG described by the Sips distribution

N(ΔG)=1π×sin(πa)e[(aRT)(ΔG°−ΔG)]1+2cos(πa)e[(aRT)(ΔG°−ΔG)]+e[(2aRT)(ΔG°−ΔG)] (3)

are equivalent to those described by the Gaussian distribution,

$$W(\Delta G)\;=\;\frac{1}{\sigma \sqrt{\pi }}\times e^{-[{\left(\Delta G^{{}^{\circ}}-\Delta G\right)}^{2}/{\left(RT\sigma \right)}^{2}]}$$ (4)

when ΔG_0_, the maximum free energy values for hapten-Ab bonding, are equivalent such that *N*(Δ*G* ^°^) = *W*(Δ*G* ^°^), thus satisfying $\sigma =2\sqrt{\pi }\left(\text{cot}\;\pi a/2\right)$.

The extent of affinity inequality among Ab subpopulations in each of the purified serum Ab preparations, which are described by the Gaussian distributions, can be measured as a ratio between the average bond strength of the strongest 5% subset and that of the weakest 5% subset. For each serum sample with average equilibrium constant (K_0_) and Gaussian heterogeneity index (σ), the weighted average of equilibrium binding constant (a) of the weakest 5% Ab was calculated as

$$\frac{\int_{10^{4}}^{a_{5th\;percentile}}\;{ae}^{{-(a-K_{0})}^{2}/\sigma^{2}}\;da}{\int_{10^{4}}^{a_{5th\;percentile}}\;e^{{-(a-K_{0})}^{2}/\sigma^{2}}\;da}$$

where Abs with affinity lower than a_5th percentile_ correspond to the lowest 5% of the population.

Similarly, the average of the strongest 5% population was calculated as

$$\frac{\int_{a_{95th\;percentile}}^{10^{10}}\;{ae}^{{-(a-K_{0})}^{2}/\sigma^{2}}\;da}{\int_{a_{95th\;percentile}}^{10^{10}}\;e^{{-(a-K_{0})}^{2}/\sigma^{2}}\;da}$$

where Abs with affinity higher than a_95th percentile_ correspond to the highest 5% of the population. Equilibrium constants for Ab binding of ligands that most closely approximate the epitope in the immunogen are rarely, if ever, found to be less than 10^4^ M^-1^ or greater than 10^10^ M^-1^, whether measured with monoclonal Abs or heterogeneous populations of purified serum Abs [1,2]. These limits were therefore taken as the boundaries of the truncated Gaussian distribution. The affinity inequalities are essentially unchanged if the boundaries are taken as 10^3^−10^10^ M^-1^ instead of 10^4^−10^10^ M^-1^. The 5^th^ and the 95^th^ percentiles of each population were calculated by first transforming the truncated Gaussian distribution to its error function, and then inverting it to calculate the respective percentiles using Matlab software.

## Results

**Fig 1:**
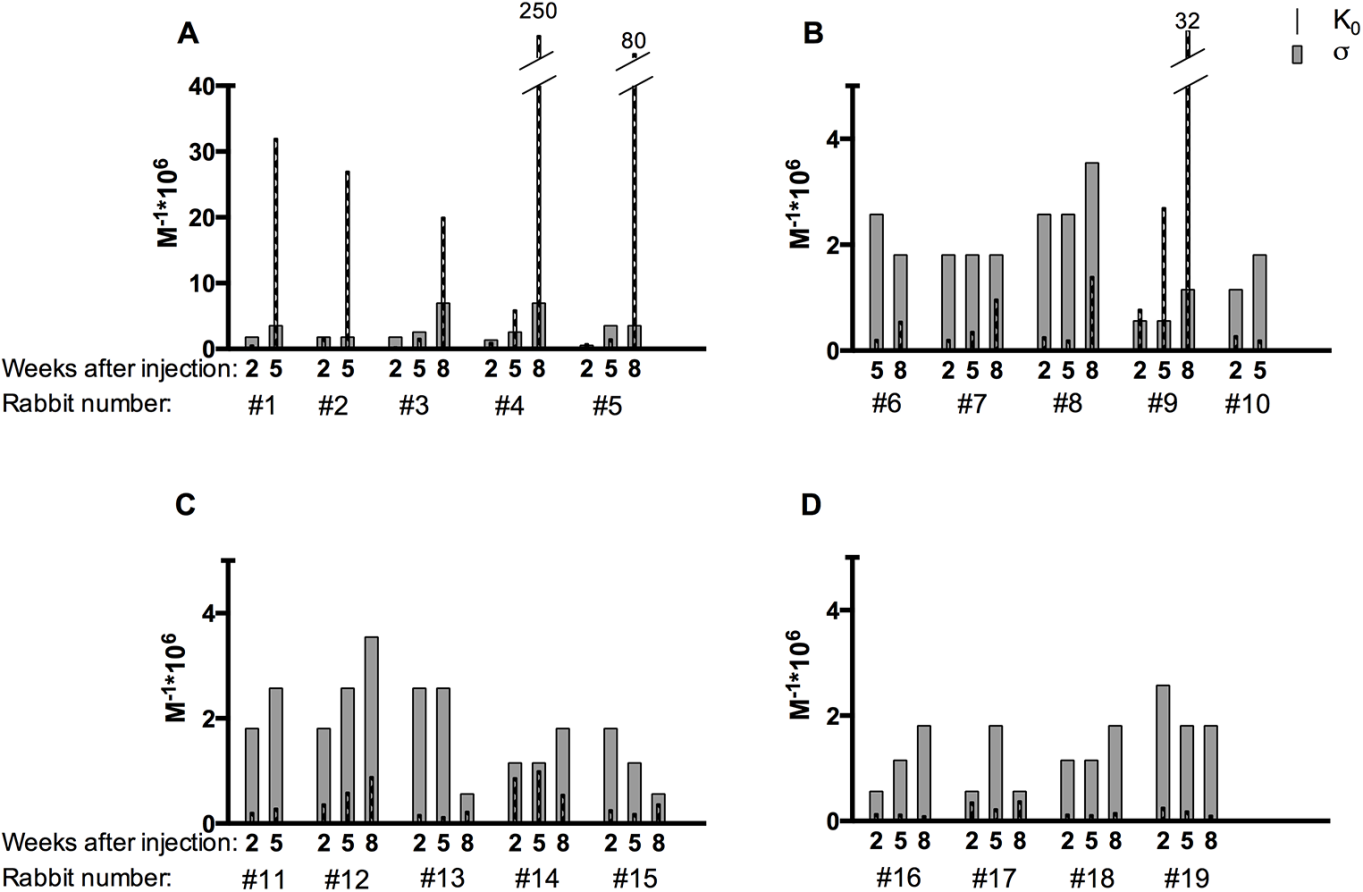
Mean affinity and Gaussian deviation of antibodies over time. (A) Mean affinity (K_0_) and Gaussian deviation (σ) (calculated from Sips to Gaussian transformation, Karush (1964)) of antibodies isolated from serum 2–8 weeks after injection of 5mg of the immunogen (2,4-DNP-bovine-γ-globulin) as incomplete Freund's adjuvant in rabbits. (B) As in (A), except 50mg injection dose. (C) As in (A), except 100mg injection dose. (D) As in (A), except 250mg injection dose.

Listed in Table 1 are average equilibrium constants (K_0_) for the binding of various Ab isolates to ε-DNP-L-lysine. ε-DNP-L-lysine approximates the principal epitope of the relevant immunogen DNP-BGG, bovine-γ-globulin with DNP groups linked to ε-amino groups of multiple lysine residues. The Abs were isolated from serum obtained at various times (2–8 weeks) from 19 rabbits injected once with various amounts of the immunogen in water-in-oil emulsion as “incomplete” Freund’s adjuvant. For those that received the smallest dose (5mg, Fig 1A), the mean affinity (K_0_) rose progressively and heterogeneity with respect to affinity also increased; Values of *a* in the Sips distribution tended to decrease and Gaussian σ to increase. However, the mean affinity (K_0_) increased faster. Hence, the coefficient of variance (σ/K_0_ ratio) decreased progressively over the 2–8 week period. Correspondingly, the ratios between the strongest and the weakest binding subsets also decreased: at the earliest time (2 weeks) this ratio was largest (the highest affinity subsets exhibited ≈13-55-fold higher K_0_ than that of the weakest subsets) and it then fell progressively to where at 8 weeks the ratio was close to 1.0. Thus, although the affinity heterogeneity index (*a* or σ) tended to increase, affinity inequality decreased over time indicating a decreasing effective diversity in Abs.

**Table 1:** Affinity inequality of antibodies after single injection of 5mg dose of the immunogen in rabbits.

Dose(mg)	RabbitNo.	Weeks fromantigeninjection	Avg.equilibriumconstant (K_0_)(M^-1^ X 10^6)	Heterogeneity index		Coefficient ofvariation ($\frac{\sigma }{K_{0}}$)	Average ofthe lowest 5thpercentile(M^-1^ X 10^6)	Average ofthe highest 5th percentile(M^-1^ X 10^6)	Affinityinequality
				Sips(a)(M^-1^ X 10^6)	Gaussian(σ* ^†^)(M^-1^ X 10^6)				
5	#1	2	**0.60**	0.70	1.8	**3.0**	0.070	3.4	**49**
"		5	**32**	0.50	3.5	**0.11**	27	37	**1.4**
5	#2	2	**1.6**	0.70	1.8	**1.1**	0.15	4.3	**29**
"		5	**27**	0.70	1.8	**0.067**	24	30	**1.3**
5	#3	2	**0.32**	0.70	1.8	**5.6**	0.059	3.2	**54**
"		5	**1.6**	0.60	2.6	**1.6**	0.14	5.5	**39**
"		8	**20**	0.30	7.0	**0.35**	9.9	30	**3.0**
5	#4	2	**1.0**	0.80	1.2	**1.2**	0.10	2.7	**27**
"		5	**5.9**	0.60	2.6	**0.44**	2.2	9.7	**4.4**
"		8	**250**	0.30	7.0	**0.028**	240	260	**1.1**
5	#5	2	**0.78**	0.90	0.56	**0.72**	0.12	1.6	**13**
"		5	**1.5**	0.50	3.5	**2.4**	0.14	7.0	**50**
"		8	**80**	0.50	3.5	**0.044**	75	85	**1.1**

* σ derived from sips to Gaussian distribution transformation by Karush (1964) equation

† Standard deviation is σ/ $\sqrt{2}$

Antibodies were isolated from serum after single injection of 2, 4-DNP-bovine-γ-globulin as incomplete Freund's adjuvant in rabbits. Numbers are rounded up to two significant figures (except for data from rabbit # 4 at 8 weeks).

When higher doses (50, 100 or 250mg) of the same immunogen were used to initiate responses, the mean K_0_ values were also initially low and affinity heterogeneity values were high (Table 2). But in contrast to the responses to the 5mg dose, in 13 of 14 rabbits that received the higher doses, these initial parameters did not change over the 2–8 week period. It is especially notable that affinity inequality remained high and changed little over at least 8 weeks. It is possible that the one exception, rabbit # 9, may not have received the full 50 mg dose.

**Table 2:** Affinity inequality of antibodies after injection of high dose of the immunogen in rabbits.

Dose (mg)	Rabbit No.	Weeks from antigen injection	Avg. equilibrium constant (K_0_) (M^-1^ X 10^6)	Heterogeneity index		Coefficient of variation ($\frac{\sigma }{K_{0}}$)	Average of the lowest 5th percentile (M^-1^ X 10^6)	Average of the highest 5th percentile (M^-1^ X 10^6)	Affinity inequality
				Sips(a) *(*M^-1^ *X10^6)*	Gaussian(σ* ^†^) *(*M^-1^ *X 10^6)*				
50	#6	5	0.21	*0*.*60*	2.6	**12**	0.072	4.4	**61**
"		8	0.55	0.70	1.8	**3.3**	0.067	3.4	**51**
50	#7	2	0.21	0.70	1.8	**8.6**	0.055	3.1	**56**
"		5	0.36	0.70	1.8	**5.0**	0.060	3.2	**53**
"		8	0.97	0.70	1.8	**1.9**	0.090	3.7	**41**
50	#8	2	0.26	0.60	2.6	**9.9**	0.074	4.4	**59**
"		5	0.20	0.60	2.6	**13**	0.072	4.4	**61**
"		8	1.4	0.50	3.5	**2.5**	0.14	6.9	**49**
50	#9	2	0.78	0.90	0.56	**0.72**	0.12	1.6	**13**
"		5	2.7	0.90	0.56	**0.21**	1.9	3.5	**1.8**
"		8	32	0.80	1.2	**0.036**	30	34	**1.1**
50	#10	2	0.28	0.80	1.2	**4.1**	0.044	2.1	**48**
"		5	0.20	0.70	1.8	**9.0**	0.055	3.1	**56**
100	# 11	2	0.21	0.70	1.8	**8.6**	0.055	3.1	**56**
"		5	0.29	0.60	2.6	**8.9**	0.075	4.5	**60**
100	#12	2	0.37	0.70	1.8	**4.9**	0.060	3.2	**53**
"		5	0.59	0.60	2.6	**4.4**	0.085	4.7	**55**
"		8	0.89	0.50	3.5	**4.0**	0.11	6.4	**58**
100	#13	2	0.17	*0*.*60*	2.6	**15**	0.071	4.4	**62**
"		5	0.13	0.60	2.6	**20**	0.070	4.4	**63**
"		8	0.23	0.90	0.56	**2.4**	0.030	1.2	**40**
100	#14	2	0.87	0.80	1.2	**1.3**	0.081	2.7	**33**
"		5	1.0	0.80	1.2	**1.2**	0.096	2.8	**29**
"		8	0.55	0.70	1.8	**3.3**	0.067	3.4	**51**
100	#15	2	0.26	0.70	1.8	**6.9**	0.057	3.2	**56**
"		5	0.19	0.80	1.2	**6.1**	0.041	2.1	**51**
"		8	0.37	0.90	0.56	**1.5**	0.039	1.3	**33**
250	# 16	2	0.14	0.90	0.56	**4.0**	0.027	1.1	**41**
"		5	0.13	0.80	1.2	**8.9**	0.040	2.1	**53**
"		8	0.10	0.70	1.8	**18**	0.052	3.1	**60**
250	#17	2	0.36	0.90	0.56	**1.6**	0.039	1.3	**33**
"		5	0.23	0.70	1.8	**7.9**	0.056	3.2	**57**
"		8	0.38	0.90	0.56	**1.5**	0.040	1.3	**33**
250	#18	2	0.13	0.80	1.2	**8.9**	0.040	2.1	**53**
"		5	0.12	0.80	1.2	**9.6**	0.039	2.1	**54**
"		8	0.16	0.70	1.8	**11**	0.054	3.1	**57**
250	#19	2	0.26	0.60	2.6	**9.9**	0.074	4.5	**61**
"		5	0.19	0.70	1.8	**9.5**	0.055	3.1	**56**
"		8	0.11	0.70	1.8	**16**	0.053	3.1	**58**

* σ derived from sips to Gaussian distribution transformation by Karush (1964) equation

† Standard deviation is σ/ $\sqrt{2}$

Antibodies were isolated from serum after single injection of 2, 4-DNP-bovine-γ-globulin in 50, 100 or 250mg as incomplete Freund's adjuvant in rabbits. Numbers are rounded up to two significant figures.

## Discussion

In view of the wide (≈million-fold) range of affinity values and the affinity heterogeneity indices previously measured for Ab-Ag and Ab-hapten reactions [1,2], one may have expected that the affinity values of Abs to a defined epitope, isolated from a given serum sample, would also extend over a wide range, and indeed they do. But, importantly, the affinity inequality, measured as the ratio of the strongest and weakest binding Ab subsets, exhibits a somewhat unexpected behavior. In a comprehensive set of changes over time in response to various Ag doses, the affinity of the strongest subset was only about 10-to 30- fold higher than the weakest in early bleeds taken two weeks after a small dose of Ag injection. This limited inequality then decreased progressively to the point where at eight weeks after immunization the difference between these subsets was essentially not detectable. The observed progressive reduction in the effective affinity inequality is in accord with the view that GC B cells are in competition for limiting amounts of Ag, and evolution occurs in the strong selection limit. Note, however, that from the data (collected almost 50 years ago) we cannot comment on the evolution of the heterogeneity of clones during GC reactions. Following low Ag dose (e.g. 5mg in Table 1, Fig 1A), B cells with high affinity BCR are preferentially selected over B cells with low affinity BCR to mature into Ab-secreting plasma cells and memory B cells. For high Ag doses, there is a surfeit of Ag, and even B cells with low affinity BCR can also be stimulated to internalize sufficient antigen and receive T cell help from a potentially less limiting amount of cognate T helper cells. Thus, most B cells can mature into Ab producing plasma cells and memory B cells. High Ag dose corresponds to conditions of weak selection. While there is a dearth of information on how the actual levels of Ag on FDC depends upon dose, the two quantities are likely to be proportional. In Table 1, Table 2 and Fig 1 the dose effect is strikingly clear because the same Ag was given to all rabbits in the same way.

Though high Ag doses, like those in Table 2, are not now used in clinical vaccines, and rarely used experimentally, they are nevertheless of interest because they provide a glimpse of the range of affinities of BCR on the naïve B cells that initially respond to immunogens. Thus in rabbits that received 100–250mg Ag (Table 2), the affinities of their serum Abs were on average ≈10^5^ M^-1^ and ranged from ≈10^4^−10^5^ M^-1^ at the lower level to ≈10^6^ M^-1^ at the upper level. Values in the former range are similar to those reported for IgM monoclonal antibodies whose intrinsic univalent affinities have been carefully measured [8,34]. These low affinity levels may well be characteristic of BCRs on the naïve IgM+ naïve B cells that are first activated by immunogens and initiate formation of GC [17,20].

In the haptenated proteins used as immunogens for studies of Ab-hapten interactions and affinity maturation, there typically are many haptenic groups per protein molecule (e.g. ≈50 DNP groups attached to a protein molecule with ≈70 lysine residues). It has therefore been suggested that the haptenated epitopes are actually diverse, despite having a common haptenic group, and that this diversity could account for the affinity heterogeneity observed with a simple ligand, such as ε-DNP-L-lysine, which only approximates the actual epitopes. This possibility has been evaluated with an immunogen, ε-41-DNP-ribonuclease, in which the DNP group was attached to the epsilon amino group of lysine 41; Abs from rabbits immunized with this mono-epitope Ag were just as heterogeneous with respect to affinity for ε-DNP-L-lysine as those Abs elicited with conventional haptenated Ags [35]. The Abs elicited with another single-epitope immunogen, DNP-lysine attached to the single SH group of papain, were also heterogeneous in terms of their diverse L chains, though their affinities were not determined [36].

It is important to note that affinity has its limits as a measure of diversity: Ab molecules with the same affinity for a given ligand could differ markedly in on- and off-rates and have different paratopes and cross-reactivities with structural variants of a ligand. In contrast to the historic data presented here, which was collected by equilibrium dialysis, recent techniques such as mutation analyses in Ig genes and Ig repertoire sequencing can trace the breadth of Abs raised against Ag as the immune response progresses, quantifying how the diversity of mutations correlates with affinity [37]. Thus, the micro-evolution in GC towards less affinity inequality seen in responses to low Ag dose (Table 1, Fig 1) tells us more about forces driving B cell selection in GCs than about the extent of cross reactions and protective efficacy of affinity matured serum Abs. Indeed, the broadly-neutralizing monoclonal Abs, such as VRC01, isolated from persons persistently infected with HIV-1, are widely cross-reactive with structural variants of the epitope in many different strains of the virus despite being homogenous with respect to their affinity for a viral epitope [38]. In addition, low intrinsic affinity IgM (immunoglobulin M), which have ten binding sites per molecule, can bind strongly with high avidity to Ags that have multiple repeats of closely spaced cognate epitopes as, for example, on pneumococcal polysaccharides and influenza virus hemagglutinin. These IgM Abs have been found to protect mice against otherwise lethal influenza virus infection [39].

The measure of affinity inequality used here is based upon the assumption that a normal probability distribution function (Sips or Gaussian) is an apt description of the diversity of bond-strengths or equilibrium constants that characterize the binding of epitopes by a heterogeneous population of Ab molecules isolated from serum by immune precipitation or adsorption. This assumption is supported by agreement between theoretical curves based upon normal distribution functions and measured K values [6–8,28]. It does not mean, of course, that other distribution functions may not also be applicable. However, the only report of non-Gaussian distributions of which we are aware is based on Ab affinity measurements made with crude globulin fractions of antisera [40]; the skewed distributions bordering on bimodality may have been due to low affinity Abs produced by B cells that differentiate into Ab secretors outside of GCs, or even perhaps to non-Ig proteins in the crude globulin fractions [41].

It may well be that the affinity diversity of the Abs produced by a small number of plasma cells emerging from a single lymph node or a few GC would not correspond to a normal distribution. Nevertheless, the average affinity values (K_0_) considered here (Tables 1 and 2, Fig 1) were obtained by analyzing samples of Ag-specific precipitable Abs that each contained about 10^20^ ~10^21^ purified Ab molecules under approximation that all Abs are 7S-IgG. Thus, these data represent the average of the output of a large number of plasma cells arising from a great many GCs. Given these conditions, normal distributions (Sips or Gaussian) remain the most useful means for describing the affinity diversity of serum Abs that recognize a particular epitope.
